# The Presence of Another Person Influences Oscillatory Cortical Dynamics During Dual Brain EEG Recording

**DOI:** 10.3389/fpsyt.2020.00246

**Published:** 2020-04-17

**Authors:** Max J. Rolison, Adam J. Naples, Helena J. V. Rutherford, James C. McPartland

**Affiliations:** Child Study Center, Yale School of Medicine, New Haven, CT, United States

**Keywords:** EEG, resting state, dual brain, social cognition, interactive social neuroscience, autism spectrum disorder

## Abstract

Humans are innately social creatures and the social environment strongly influences brain development. As such, the human brain is primed for and sensitive to social information even in the absence of explicit task or instruction. In this study, we examined the influence of different levels of interpersonal proximity on resting state brain activity and its association with social cognition. We measured EEG in pairs of 13 typically developing (TD) adults seated in separate rooms, in the same room back-to-back, and in the same room facing each other. Interpersonal proximity modulated broadband EEG power from 4–55 Hz and individual differences in self-reported social cognition modulated these effects in the beta and gamma frequency bands. These findings provide novel insight into the influence of social environment on brain activity and its association with social cognition through dual-brain EEG recording and demonstrate the importance of using interactive methods to study the human brain.

## Introduction

Social interaction is central to human experience and necessary for normative brain development. The presence of another person is environmentally salient, drawing attention and neural resources (1). During development, such social interactions provide required information to experience-expectant brain systems supporting specialization of a network of brain regions for processing social information (2, 3), and it is hypothesized that primate brains evolved to support complex social cognition (4, 5). Thus, in addition to actively supporting social performance, this network remains engaged even when a person is “at rest” rather than engaged in an explicitly social activity (6).

The association between resting state brain activity and social cognition is incompletely understood. Neuroimaging studies consistently implicate atypical resting activity across multiple modalities in clinical populations with impaired social cognition (7–10). Even in nonclinically ascertained populations, EEG studies have identified alterations in power in the alpha frequency range (8–13 Hz) associated with social cognition (11). Despite strong evidence for an association between at-rest brain activity and social function, the majority of research has measured brain activity when participants are in isolation in an EEG recording chamber, MRI, or MEG; we know little about brain activity during *in vivo* social interactions. Interactive social neuroscience (12), or second person neuroscience (13), the study of brain function during live social interaction, seeks to measure brain activity in a more ecologically valid manner.

Increasing efforts have focused on using EEG hyperscanning to understand the neural basis of social interactions, with protocols being developed to allow this approach to be more widely implemented across research groups (14). EEG hyperscanning during cooperative games reveals variability in the activity of different frequency bands in prefrontal areas (15), with activity in prefrontal and anterior cingulate regions differentiating player order during card games (16). EEG hyperscanning has also evidenced value during cooperative (17–19) and competitive social interactions (20, 21). Additionally, EEG hyperscanning has demonstrated interpersonal synchrony when people are performing coordinated movements (22–26). Importantly, correlations between participants’ EEG activity may be shaped by a host of individual differences, including empathy, social closeness, and autistic traits (27, 28). Clinically, hyperscanning approaches may also be especially relevant to our understanding of the neural basis of autism (12). Using fNIRS, children with ASD evidenced variability in neural synchronization in frontal areas when interacting with their parents compared to when they were completing the task alone under parental observation or during a no interaction comparison condition (29).

Using these methods, researchers have identified task-related differences (28) and differences in the alpha, beta, and theta frequency bands when participants were together versus alone, which were modulated by anxious attachment style (30), but relationships with resting brain activity and social performance remain unexplored.

In this study, we examined how the presence of another person modulated resting state brain activity. We recorded EEG simultaneously from pairs of participants during three social contexts: in separate rooms, together seated back-to-back, and together facing each other. EEG data was recorded when participants had their eyes open and their eyes closed across the three social contexts. We predicted that variation in social context would alter resting-state oscillatory brain activity. Specifically, we expected that alpha would be sensitive to changing social dynamics based on the well-established evidence that alpha indexes vigilance and arousal, as well as prior work demonstrating an association between alpha activity and autistic traits (11). Additionally, we expected that variation in oscillatory activity between contexts, as a marker of sensitivity to social context, would be associated with social cognition, as measured through self-report of social ability.

## Methods

### Participants

Twenty college-aged participants from the New Haven community (M=21.7 years, SD=0.45, 6 male) participated in 10 same-sex dyads (recruited independently and paired arbitrarily). Exclusionary criteria included prescription medications affecting cognitive processes (including benzodiazepines, barbiturates, antiepileptics, carbamazepine, and valproic acid), history of head trauma or serious brain or psychiatric illness, or history of learning or intellectual disability. All procedures were conducted with the understanding and written consent of participants and with approval of the Human Investigations Committee at the Yale School of Medicine. Participants were compensated for their participation in the study.

### Behavioral Measures

Participants completed a series of self-report questionnaires designed to measure variation across subclinical to clinical levels of social and communicative performance and impairment: the Social Responsiveness Scale 2^nd^ Edition (31) and the Broad Autism Phenotype Questionnaire (32).

### EEG Procedures

#### Task

**Figure 1:**
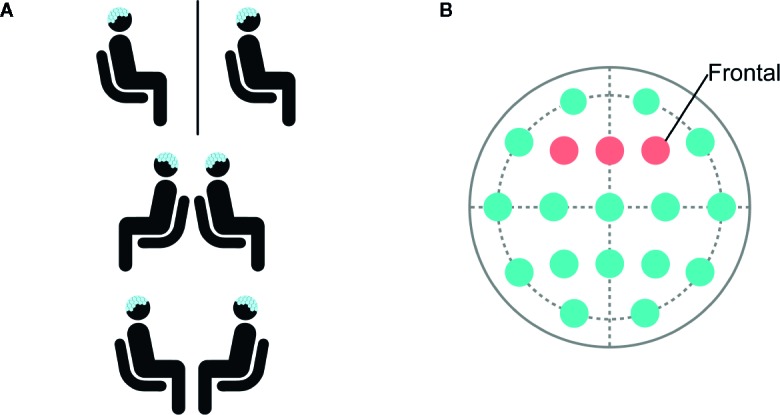
**(A)** Participants were seated in separate rooms, back-to-back, and facing each other. **(B)** Data was selected and analyzed from frontal electrodes F3, Fz, and F4.

Following separate consenting procedures, participants were introduced to one another and seated in the same room for EEG application. During EEG recording, participants sat quietly for two minutes in two eye orientations (eyes opened (EO) or eyes closed (EC)) across three conditions: (1) “separate” rooms, (2) the same room “back-to-back”, and (3) the same room “facing” each other (**Figure 1A**). During EC across all three social contexts, participants were instructed to remain still with their eyes closed. During EO, when participants were in separate rooms and back-to-back, they were instructed to remain still and pick a point straight ahead and fixate on the point. When participants were facing, they were instructed to remain still while looking into each other’s eyes. Therefore, the facing EO condition demonstrated joint-gaze. While a fixed order precludes estimation of order effects, a full counterbalancing of experimental conditions was not possible with the planned sample size. Moreover, in order to draw comparisons between the current study and prior studies of resting-state EEG recorded in isolation, we similarly began by recording in separate rooms. Additionally, we speculated that the novelty of the face-to-face condition would limit the interpretation of subsequent conditions. For these reasons, we adopted this fixed order of social context administration.

EEG was recorded using the B-Alert X-24 20 channel wireless EEG sensor net (Advanced Brain Monitoring Inc., Carlsbad CA). Continuous EEG data was recorded at 256 Hz using B-Alert acquisition software [Version 2.05.05; (33)] with joint mastoid reference. Electrode impedance was kept under 10 kOhms with Synapse Conductive Electrode Cream. Continuous EEG data across systems was synchronized using a pair of ABM External Sync Units (ESU) connected to the stimulus presentation computer *via* a split cable TTL pulse. An audio tone signaling the start and end of each condition was presented using E-Prime 2.0 (34). EEG was marked every 1000 milliseconds during each condition.

#### EEG Processing

EEG was filtered from 0.5 to 100 Hz and preprocessed using EEGLAB (35). Data was selected from frontal electrodes due to the importance of the frontal cortex in modulating attention (**Figure 1B**). PREP pipeline (36) was used to remove line-noise, detect, and interpolate bad channels. Next, independent component analysis (ICA) was performed and eye-blink components were manually identified based on scalp topography and removed. Data was epoched into 1,000-ms segments. Artifact detection was performed with a 40-µV threshold using a 50-ms moving window in 25-ms steps, and epochs containing artifact were rejected. Participants with more than 50% rejected epochs per category were excluded from analyses. Included participants had an average of 7.2% rejected epochs.

Frequency decomposition was performed using the Fieldtrip Toolbox (37). Theta (4–7 Hz), alpha (8–12 Hz), beta (12–24 Hz), and gamma (30–40 Hz) frequency bands were defined based on prior studies (30). Epochs were zero padded to contain 25,600 samples, mean detrended, windowed with a Hann window, and power was calculated using a multitaper fast Fourier transform (FFT) with four tapers.

#### Analyses

Data from 13 participants was included in analyses following artifact detection. Parametric data was analyzed using repeated measures analysis of variance (ANOVA), and data not meeting criteria for normality as indicated by Shapiro-Wilk’s test was analyzed using Friedman’s 2-way ANOVA by Rank. EEG power in the theta and gamma frequency ranges was analyzed separately using 3 (separate/back-to-back/facing) × 2 (EO/EC) repeated measures ANOVA. EEG power in the alpha and beta frequency ranges was analyzed separately using Friedman’s two-way ANOVA by rank for eye orientation and condition. Planned comparisons were performed to investigate directionality of observed effects, utilizing paired samples *t* tests for parametric data and Wilcoxon Signed Rank test for nonparametric data. Spearman’s rank correlations were used for assessing the relationship between changes in EEG power and social function. Difference scores were calculated by subtracting the absolute power between different conditions (SEP-BACK, BACK-FACE) For all analyses, the statistical significance level was set at α < 0.05, and Bonferroni correction was applied to correct for multiple comparisons. Effect size estimates for analyses of variance, t-tests, and behavioral correlations were calculated with partial eta-squared (η^2^_partial_), Cohen’s d (d), and Spearman’s rank correlation coefficient (*ρ,* rho), respectively. Confidence intervals (CI) for Spearman’s rank correlations were calculated based on the Fisher r-to-z transformation.

## Results

### Theta Power

**Figure 2:**
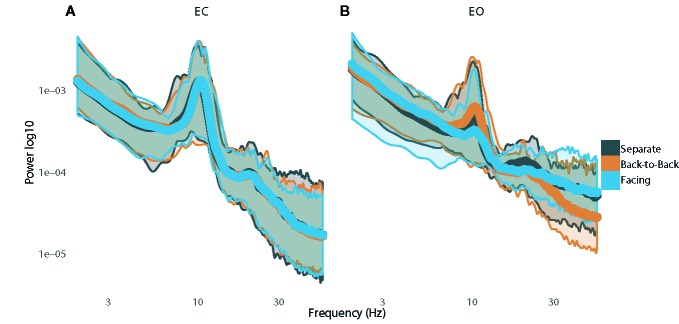
Plots of power spectra with standard error while resting with **(A)** eyes closed and **(B)** eyes open.

**Figure 3:**
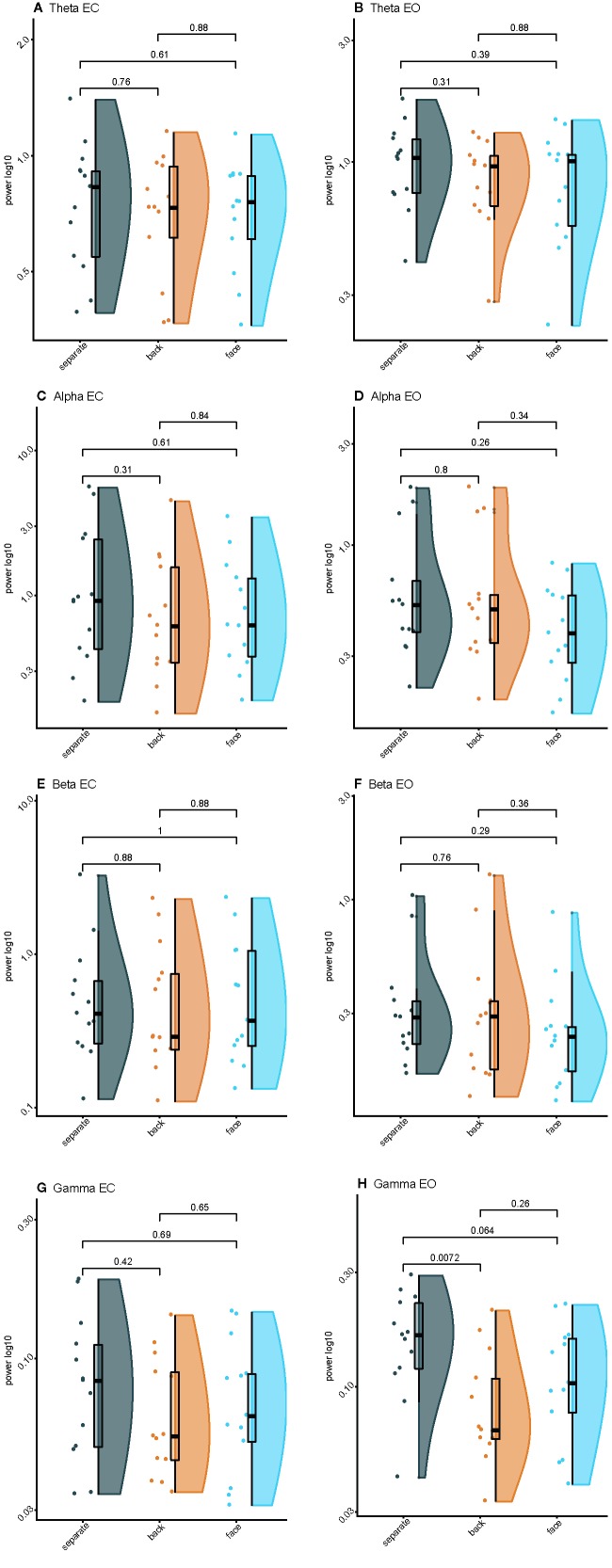
Raincloud plots of spectral power for varying levels of interpersonal proximity: **(A)** Eyes closed (EC) theta power; **(B)** Eyes opened (EO) theta power; **(C)** EC alpha power; **(D)** EO alpha power; **(E)** EC beta power; **(F)** EO beta power; **(G)** EC gamma power; **(H)** EO gamma power.

Spectral plots are shown in **Figure 2**. Results revealed a main effect of eye orientation on theta power, F(1,12)=6.6, p=0.03, η^2^_partial_=0.35, with participants demonstrating greater theta-band activity during EO relative to EC. Furthermore, there was a main effect of condition, F(2,24)=4.2, p=0.03, η^2^_partial_=0.26, indicating that theta activity was greater when separate compared to back-to-back, p=0.01, or facing, p=0.04 (**Figures 3A, B**). There was no interaction between eye orientation and condition, p=0.48.

### Alpha Power

Results revealed greater alpha activity during EC than EO when resting separately Z=−2.7, p < 0.01, back-to-back, Z=−2.2, p=0.03, and facing, Z=−3.2, p < 0.01. Additionally, there was an effect of condition during EC, χ^2^(2)=11.2, p < 0.01, such that alpha activity was greater when back-to-back compared to when resting separately, Z=1.3, p < 0.01. There was no effect of condition during EO, χ^2^(2)=3.2, p=0.20 (**Figures 3C, D**).

### Beta Power

Results revealed greater beta activity during EC than EO when resting separately, Z=−3.0, p < 0.01, back-to-back, Z=−3.0, p < 0.01, and facing, Z=−3.1, p < 0.01. There was no effect of condition during EC, χ^2^(2)=1.1, p=0.58. However, there was an effect of condition during EO, χ^2^(2)=12.2, p < 0.01, such that beta activity was greater during joint-gaze while facing compared to resting separately, Z=1.3, p < 0.01, or resting back-to-back, Z=1.0, p=0.03 (**Figures 3E, F**).

### Gamma Power

A significant interaction between eyes and condition, F(2,24)=5.9, p < 0.01, η^2^_partial_=0.33, revealed that gamma activity was greater during EO than EC when separate, t(12)=4.7, p < 0.01, d=1.25, and when facing, t(12)=3.3, p < 0.01, d=0.84, but was not different when back-to-back, t(12)=1.6, p=0.13. During EO, gamma activity was greater when resting separately than resting back-to-back, t(12)=4.2, p < 0.01, d=1.23, or facing with joint-gaze, t(12)=2.4, p=0.03, d=0.73. However, there was no difference between resting back-to-back or facing with joint-gaze during EO, t(12) =−2.0, p=0.08. During EC, gamma activity was greater when resting separately than resting back-to-back, t(12)=2.5, p=0.03, d=0.51. Gamma activity was not different when resting separately versus resting while facing, t(12)=2.1, p=0.06, or back-to-back versus facing, t(12) =−1.3, p=0.21 (**Figures 3G, H**).

### Behavioral Correlations

**Figure 4:**
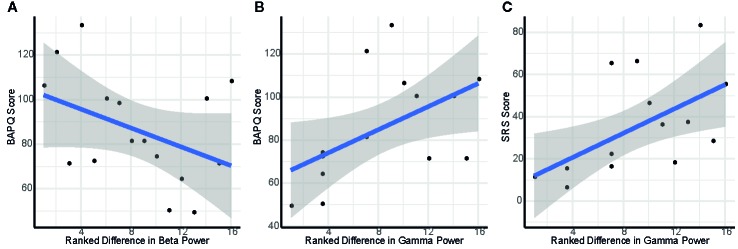
Scatterplots depicting association of self-reported social cognition and variation in interpersonal proximity: **(A)** Ranked difference in eyes closed (EC) beta power between facing versus back-to-back and BAPQ score; **(B)** Ranked difference in EC gamma power between separate and back-to-back and BAPQ score; **(C)** Ranked difference in EC gamma power between separate and back-to-back and SRS score.

Greater difference in EC beta power when back-to-back versus facing was associated with lower scores on the BAPQ, *r*=0.60, *p*=0.03, 95% CI [0.074, 0.865] (**Figure 4A**).

Higher total score on the BAPQ was associated with greater difference in EC gamma activity between separate and back-to-back, *ρ*=0.61, p=0.03, 95% CI [0.089, 0.868] (**Figure 4B**). Additionally, this difference score between separate and back-to-back was associated with higher total score on the SRS, *ρ*=0.70, p < 0.01, 95% CI [0.243, 0.902] (**Figure 4C**).

## Discussion

The current study recorded resting-state EEG simultaneously from two adults while social context was manipulated–with participants separated, in the same room but back-to-back, or in the same room and facing one another. Differential oscillatory power in the theta, alpha, and gamma bands was observed when participants were isolated; when in the presence of another person, facing towards one another or away from one another did not influence resting neural activity. These results suggest that the social presence of another human, regardless of interpersonal orientation, modulates brain activity. We interpret these findings as suggestive of the adoption of an “interpersonally-oriented stance” when in proximity to a potential social partner. The activity was not modulated by facing towards or away from the potential partner which suggests that without an explicit social task, default mode activity is tuned to the presence of another person rather than more granular levels of information, such as face-to-face orientation. Specifically, theta, alpha, and gamma activity attenuation in the presence of another person suggest that activity in these frequency bands may be suppressed in preparation for social interaction.

Additionally, theta and gamma activity was greater when resting with eyes open, while alpha and beta power was greater while resting with eyes closed. These results are consistent with prior studies demonstrating a balance of excitatory and inhibitory activity with a U-shaped profile (38).

Greater difference in beta activity between being back-to-back and facing another person with eyes closed was associated with better self-reported social function. These findings suggest that greater sensitivity to differences in the social environment may contribute to better social cognition. Within the gamma band, differential neural response to isolation versus presence of another person was associated with self-reported social function. Specifically, a greater difference in gamma activity between separate and back-to-back was associated with more impaired self-reported social function. Gamma activity has been associated with social cognition and mentalizing (39), suggesting a relationship between neural attunement to conspecifics and social performance. Specifically, it has been hypothesized that gamma activity is associated with the integration of sensory with socially and emotionally salient information (39, 40), as well as with emotional regulation (41). Additionally, gamma activity has been associated with brain-to-brain synchronization during social interactions (42). Our finding of a relationship between gamma activity and social function aligns with prior research demonstrating correlated resting gamma activity in familiar, but not unfamiliar, dyads (42). These results add to a nascent literature showing relationships among psychological attributes and modulation of resting brain activity by the presence of another person; for example, other studies have shown this modulation to be related to attachment status (30). Given the relevance of social interaction to many clinical conditions, such as autism spectrum disorder, this study reveals a novel avenue for investigating social brain function dissociated from active social tasks.

These findings indicate the overarching influence of interpersonal proximity on resting brain activity. The observation of neural modulation based on mere presence of another person has significant implications for electrophysiological brain research on resting neural activity. Many investigations presume that resting state brain activity represents a task-free “absolute” baseline. Our findings demonstrate that the social environment influences baseline brain activity, suggesting that methodological variation, such as the presence of an examiner in the room, may exert significant influence on results. These findings add to a growing literature demonstrating the importance of studying the brain during social interaction across a variety of contexts. In particular, EEG hyperscanning offers promise for the investigation of these questions because EEG is relatively scalable, cost-effective, and produces a robust signal (12, 14, 43).

Several limitations of the present study should be addressed in future research. Our sample size was limited and precluded more complex and exploratory analytic approaches, such as whole scalp analyses, functional connectivity, and interbrain synchrony. Replication and a more comprehensive analytic approach will be required in future studies with larger samples. Although our analyses do not examine interbrain synchronization between dyads, we consider the simultaneous recording an important part of the experimental procedure in that both participants were in comparable circumstances (e.g., both observed), which would not be the case with only one recording device (e.g., observer and observed). We felt that this arrangement was necessary to be consistent with a naturalistic social interaction. Because we utilized a fixed order for conditions, we were not able to fully explore the effects of a changing social context. Since we utilized only same-sex dyads we could not examine the influence of sex on interpersonal modulation of brain activity. We could not monitor eye gaze during our face-to-face condition; given the importance of eye contact, use of eye-tracking would enable more nuanced investigation of the influence of eye contact during face-to-face interactions. Additionally, gamma activity has been associated with a multitude of cognitive processes, as well as eye movements, thus unexplored factors may contribute to the observed effects.

## Conclusions

The current study recorded resting-state EEG simultaneously from two adults in varying social contexts to investigate the influence of the social environment on baseline brain activity. Results reveal modulation of brain activity based on varying levels of interpersonal proximity, specifically in the theta, alpha, and gamma frequency bands. This study adds to a growing body of evidence suggesting that resting state brain activity is strongly subject to the influence of social context and that these differences in resting state brain activity are associated with social cognition. Our findings provide new insight into resting state neural dynamics and further emphasize the utility of interactive social neuroscience approaches for the study of varying brain states.

## Data Availability Statement 

The datasets generated for this study are available on request to the corresponding author.

## Ethics Statement

The studies involving human participants were reviewed and approved by the Yale University Human Investigations Committee. The patients/participants provided their written informed consent to participate in this study.

## Author Contribution

MR, AN, and JM conceived of and designed the experiment. MR performed data collection. MR, AN, and HR analyzed the data. MR, AN, HR, and JM wrote the manuscript.

## Funding

This work was supported by Autism Science Foundation Student Fellowship (MR) and Research Accelerator (AN) Grants; National Institutes of Mental Health R01 MH111629, R01 MH107426, and K23 MH086785 (JM); Patterson Trust Grant 13-002909 (JM); Autism Speaks Postdoctoral Fellowship (AN); and INSAR Slifka/Ritvo Award (AN).

## Conflict of Interest

The authors declare that the research was conducted in the absence of any commercial or financial relationships that could be construed as a potential conflict of interest.
